# Supplementary material for: A Comprehensive Assessment of Ultraviolet-Radiation-Induced Mutations in Flammulina filiformis Using Whole-Genome Resequencing
Source: J Fungi (Basel). 2024 Mar 20;10(3):228. doi: 10.3390/jof10030228 (PMC10971301; doi:10.3390/jof10030228)
Supplement: Supplementary file 1 [file jof-10-00228-s001.zip › Supplementary Material S8/KEGG annotation/out/64381550635650.os/KO/out_map/map04024.html]

KEGG PATHWAY: cAMP signaling pathway - Reference pathway


|  |  |
| --- | --- |
| **cAMP signaling pathway - Reference pathway** |  |

[
Pathway menu
| Organism menu
| Pathway entry
| Show description
| User data mapping
]

cAMP is one of the most common and universal second messengers, and its formation is promoted by adenylyl cyclase (AC) activation after ligation of G protein-coupled receptors (GPCRs) by ligands including hormones, neurotransmitters, and other signaling molecules. cAMP regulates pivotal physiologic processes including metabolism, secretion, calcium homeostasis, muscle contraction, cell fate, and gene transcription. cAMP acts directly on three main targets: protein kinase A (PKA), the exchange protein activated by cAMP (Epac), and cyclic nucleotide-gated ion channels (CNGCs). PKA modulates, via phosphorylation, a number of cellular substrates, including transcription factors, ion channels, transporters, exchangers, intracellular Ca2+ -handling proteins, and the contractile machinery. Epac proteins function as guanine nucleotide exchange factors (GEFs) for both Rap1 and Rap2. Various effector proteins, including adaptor proteins implicated in modulation of the actin cytoskeleton, regulators of G proteins of the Rho family, and phospholipases, relay signaling downstream from Rap.

|  |  |
| --- | --- |
| Reference pathway | 100% |
